# Nutritional Supplementation and Exercise as Essential Allies in the Treatment of Chronic Heart Failure: The Metabolic and Molecular Bases

**DOI:** 10.3390/nu15102337

**Published:** 2023-05-16

**Authors:** Evasio Pasini, Giovanni Corsetti, Francesco Saverio Dioguardi

**Affiliations:** 1 Department of Clinical and Experimental Sciences, University of Brescia, 25100 Brescia, Italy; evpasini@gmail.com; 2 Italian Association of Functional Medicine, 20855 Lesmo, Italy; 3 Department of Internal Medicine, University of Cagliari, 09042 Cagliari, Italy; fsdioguardi@gmail.com

**Keywords:** heart failure, malnutrition, exercise, amino acids, mTOR, Deptor

## Abstract

Chronic heart failure (CHF) is one of principal health problems in industrialized countries. Despite therapeutical improvement, based on drugs and exercise training, it is still characterized by elevated mortality and morbidity. Data show that protein energy malnutrition, clinically evident primarily with sarcopenia, is present in more than 50% of CHF patients and is an independent factor of CHF prognosis. Several pathophysiological mechanisms, primarily due to the increase in blood hypercatabolic molecules, have been proposed to explain this phenomenon. Nutritional supplementation with proteins, amino acids, vitamins and antioxidants have all been used to treat malnutrition. However, the success and efficacy of these procedures are often contradictory and not conclusive. Interestingly, data on exercise training show that exercise reduces mortality and increases functional capacity, although it also increases the catabolic state with energy expenditure and nitrogen-providing substrate needs. Therefore, this paper discusses the molecular mechanisms of specific nutritional supplementation and exercise training that may improve anabolic pathways. In our opinion, the relationship between exercise and the mTOR complex subunit as Deptor and/or related signaling proteins, such as AMPK or sestrin, is pivotal. Consequently, concomitantly with traditional medical therapies, we have proposed a combination of personalized and integrated nutritional supplementation, as well as exercise to treat malnutrition, and anthropometric and functional CHF-related disorders.

## 1. The Clinical Problem

Chronic heart failure (CHF) is one of principal health problems of the industrialized countries. It is a complex syndrome where, although the “*primum movens*” is heart disease, it also affects many organs and systems of the human body [1]. Despite recent therapeutical improvements based on a combination of specific medical approaches/therapies, CHF is still characterized by elevated mortality and morbidity [2].

Interestingly, recently the benefits of cardiac rehabilitation and exercise training have been shown in patients with heart failure, including a reduction in morbidity and mortality. However, data also show that even light exercise causes a significant catabolic demolition of muscular proteins [3]. This catabolic effect of exercise is more prominent in patients with malnutrition. We must therefore specify that malnutrition is a generic term that includes two pathophysiological and clinical conditions: overnutrition and undernutrition.

We can say that surplus nutrients intake causes overnutrition, which in turn causes obesity. However, the presence of obesity does not necessarily suggest that there is no alteration of protein metabolism. Indeed, we should remember that there is a clinical condition, namely sarcopenic obesity, in which the patient is obese, but has all the characteristics of sarcopenia, including reduced muscle mass and muscle strength. Therefore, the presence of protein metabolism disarrangements should also be sought in obese patients with CHF.

On the contrary, undernutrition is due to a lack of nutrients. It can result in reduced growth or weight loss according to age and/or concomitant diseases. Undernutrition can occur either due to protein energy wasting or as a result of micronutrient deficiencies. It can be a major health problem both in children, where it is responsible for incomplete physical and mental development, as well as in adults. Interestingly, undernutrition is particularly present in elderly people and women. It should be pointed out that undernutrition is an increasing health problem in people aged over 65 years in developed countries, mainly due to physical, psychological and social factors. Indeed, aged people reduce dietary intake because they may have both physical and/or social problems, such as chewing and swallowing difficulties, depression, intestinal-related diseases, and/or poverty and loneliness.

The signs and symptoms of micronutrient deficiencies depend on which micronutrients are lacking. Basically, an inadequate intake of micronutrients includes iodine, vitamins and iron. Clinically, these can cause, respectively, hypothyroidism, hypovitaminosis and hypo-hemoglobinemia (named anemia). Interestingly, anemia is often present in patients with CHF, and it is commonly caused by iron deficiency, although other micronutrients are also needed for hemoglobin synthesis [4].

We also need to specify that iron is fundamental for other important metabolic processes of cell life, including DNA synthesis and matrix metalloprotease activities (MMPs). It should be noted that MMPs play a fundamental role in degrading both matrix and non-matrix proteins, which in turn influence tissue repair and remodel the response to injury, such as trauma or necrosis. In addition, MMPs are involved in the development of atheroma and/or chronic diseases.

We should also remember that hemoglobin is a heme protein. As such, hypo-hemoglobinemia indicates that there is a lack of other heme proteins, including fundamental proteins involved in the energy use/production (i.e., myoglobin, cytochrome-c), in the defense against oxygen free radicals (i.e., catalase, peroxidase) or in inflammatory processes (i.e., cyclooxygenase, NOS) [4].

Undernutrition is considered protein energy malnutrition when both the micronutrient deficiencies, and an imbalance of protein/energy intake and protein/energy expenditure are present. It differs from calorie restriction due to hypoalimentation, which has no negative health effects. It is characterized by symptoms such as weight loss with normal serum albumin. Notably, epidemiological findings show that protein energy malnutrition is often present in patients with CHF, along with traditional CHF symptoms [5].

These alterations cause global metabolic disfunction with protein disarrangements present objectively in CHF patients as a loss of muscular body mass/functions known as sarcopenia which can progress to cachexia [4]. It is interesting to note that protein energy malnutrition correlates with mortality regardless of heart disease severity in CHF patients [6]. However, although protein energy malnutrition has such an important clinical impact, it is still too often underestimated and/or not accurately evaluated or counteracted by most clinicians [7].

Here, based on molecular and pathophysiological evidence, we have proposed to integrate nutritional supplementation with specific molecules, with exercise training and traditional medical therapy to create a winning alliance to treat patients with CHF in the best possible manner.

## 2. Malnutrition: Quantification and Causes

Following our proposal, the first step is to identify and quantify protein energy malnutrition. As stated, it is the reduced balance between body nutrient intake by food and bodily metabolic needs [1]. Because CHF increases these metabolic needs, while nutrient intakes are reduced or remain unchanged, this malnutrition is present in a great proportion of patients with CHF [8]. Therefore, we believe that a careful clinical evaluation of patients with CHF should take into consideration both the food intake and any signs of protein energy malnutrition.

Food intake can be evaluated through questionnaires or, more accurately, day–food diaries. Patients’ signs of protein energy malnutrition could be identified looking at a loss of muscle mass and strength within different settings. Indeed, a simple measurement of anthropometric variables, such as tricipital skin-fold thickness (index of fat mass) and arm muscle area (index of lean mass), and a hand grip test can be measured in outpatients. More sophisticated and demanding techniques, such as ultrasound and/or dual X-ray absorptiometry (DXA), can also be used in hospitalized patients. Additionally, specific blood markers such as albumin, hemoglobin, lymphocytes count, red cells, transferrin and retinol-binding proteins could be measured to confirm the presence of metabolic malnutrition [9].

There are no single tests which optimally diagnose protein energy malnutrition. However, the authors’ advice is that evaluating albumin in plasma is mandatory. Indeed, this test is cheap, available in any laboratory, and if the levels fall under 3.5 g/dL, malnutrition should be suspected and great attention should be paid to those patients. Albumin has a long half-life (about 3 weeks), thus it takes time to normalize its values. However, on the contrary, if found to be low, long-term malnutrition and any other cause of hypo-albuminemia should be carefully excluded (i.e., proteinuria, blood loss, liver insufficiency, acute inflammation, etc.). In any case, there is much research showing that hypoalbuminemia strongly correlates to mortality, independent of the illness [10]. There is growing evidence to show that malnutrition, and thus hypoalbuminemia, should be treated primarily with essential amino acids (EAAs), since non-essential ones contribute to urea, but not albumin synthesis [11,12].

To monitor the short-term adequacy of therapy in very demanding conditions, the normalization of proteins marked by shorter-term syntheses would be used. If these are not successfully influenced by nutritional therapy, it indicates that the therapy is inadequate to match the needs, so the prognosis could worsen.

If there are any signs of protein energy malnutrition, it would be also helpful to identify specific nutritional deficiencies by measuring blood vitamins and ions, which are the indispensable co-factors of the enzymes which govern anabolic pathways [13,14].

The causes of protein energy malnutrition in patients with CHF, especially when elderly, is probably multifactorial. Reduced food intake due to loss of appetite, change in smell sensory function, impaired chewing due to pathologies and/or dental prostheses, social isolation, as well as intestinal malabsorption from altered mesenteric circulation and/or dysbiosis, could all be responsible for CHF-induced malnutrition [15,16].

However, the signs of protein energy malnutrition are present not only in CHF, but also in many different chronic and acute diseases such as cancer, infectious and collagen vascular disorders. All these diseases have a common finding: the increase in catabolic molecules such as hormones, and pro-inflammatory cytokines such as TNFα and IL1-6 [6].

Nowadays, it is well known that both catabolic neurohormones and inflammatory cytokines play a significant role in the progression of heart failure, inducing severe metabolic perturbations, such as insulin resistance syndrome (IRS), responsible for muscular wasting, cachexia and global metabolic disorders [17]. Indeed, IRS significantly influences the biochemistry of peripheral muscle, globular proteins and adipose tissue already compromising other causes of malnutrition, such as reduced food intake. The lack of anabolic stimulation due to IRS causes protein degradation and amino acid (AA) release firstly in the muscles, and then an alteration in globular circulating proteins. AAs are deaminated and their carbon skeleton is used: (a) in the liver, to produce glucose by gluconeogenesis and (b) in the whole body, to support the increased global energy and metabolic needs. This condition is also called hypermetabolic syndrome (HS) and causes protein disarrangements, which worsen clinical conditions of chronic diseases, including CHF [1,18].

Furthermore, IRS inhibits mRNA synthesis of phosphoenolpyruvate carboxykinase (the key enzyme of gluconeogenetic pathway), with a consequent further reduction in gluconeogenesis by pyruvate originated from lactate in the liver. This condition causes a vicious circle, where reduced nutrition does not supply enough exogenous nutrients and the liver produces glucose from AAs, so that the body’s proteins are destroyed. AAs are then released into the blood and deaminate to produce glucose, essential to maintain the energetic metabolism of fundamental structures such as the brain and erythrocytes [19].

Another consequence of the IRS is that muscle glycogen reserves are significantly reduced and lost, since free fatty acids (FFAs) become the principal fuel for the muscle. However, FFA use is a limiting factor for muscular energy production because excess FFA oxidation reduces phosphocreatine production during exercise, with a consequent skeletal muscle fatigue [19]. In addition, we should remember that insulin perturbations influence lipolysis in adipose tissue, and consequently FFA viability for acetyl-CoA indispensable for mitochondrial energy production, so that acetate originates predominately from AA oxidation [14].

Taken together, this evidence suggests that catabolic neurohormones and inflammatory cytokines are implicated in IRS found in CHF. IRS, in turn, induces protein catabolism, activates gluconeogenesis from AAs and alters lipid metabolism. These metabolic features cause protein catabolic demolition of both skeletal muscular and globular (i.e., albumin, hemoglobin, immunoglobulins, etc.) proteins, such as AAs, and are consumed mostly to support the increased global energetic needs, rather than being available for protein synthesis and function, with consequent proteinic metabolism disarrangements, which are clinically evident in sarcopenia and worsen the patient prognosis [20].

## 3. Integrated Therapeutical Strategies: Exercise Training and Nutritional Supplementation

In our opinion, evidence suggests that exercise training and nutritional supplementation, combined with traditional therapy, are promising integrated therapeutic strategies to treat CHF patients for the following reasons:

### 3.1. Exercise Training

It is well established that aerobic resistant exercise training stimulates anabolic stimuli, thus reducing muscular wasting, mortality and hospitalization, and improving metabolism in CHF patients. However, the mechanism responsible for these beneficial effects has not been fully explained. Recently, different mechanisms have been identified and extensively reviewed [21].

It has been demonstrated that both light exercise and aerobic exercises, and/or overall fitness maintain telomere length. This is a very interesting phenomenon because telomere length controls the terminal regions of chromosomal DNA from progressive degradation, and ensures the integrity of linear chromosomes, correlating with cellular damage including senescence-induced damage [21].

Other studies show that physical activity is strongly correlated with epigenetic changes. Indeed, exercise influences DNA methylation and consequently transcription or the repression of genes, which influence cell proliferation [21]. In addition, metabolomic studies show that physical exercise modifies thousands of blood metabolites, which are in turn able to influence, global metabolism and inflammation [21]. However, one question remains unresolved: what type of physical activity/exercise has the greatest health promoting effects? Clinical data show that physical activities have pharmacological-like effects, called “gymno-mimetics”, which is why aerobic exercise for CHF patients is recommended by the current European Society of Cardiology (ESC) guidelines (class I, level of evidence A) [22,23], as well as regular excise and physical activities being inserted into the guidelines for the maintenance of general health by the World Health Organization [24].

However, data also show that even light exercise causes a significant release of muscular AAs, suggesting the catabolic demolition of muscular proteins, at least in untrained CHF patients [3]. We believe that it is possible to reduce these catabolic effects of exercise-stimulated protein anabolism by matching exercise and nutritional intakes, so that nutritional supplementation and exercise could became allies in the treatment of CHF.

### 3.2. Nutritional Supplementation

As mentioned earlier, data suggest that CHF-induced hypercatabolic syndrome causes AA metabolism alterations and the protein disarrangement of both globular and muscular proteins. As a result, the exogenous supplementation of food preproteins and/or free AAs could be a valid therapeutical strategy to use with CHF patients. However, data also show that improvement of the metabolic and nutritional status of muscle-depleted CHF patients occurs only when adequate energy protein intake is combined with a specific mixture of all free forms of essential AAs (EAAs) in a stoichiometric ratio, and not with a simple increase in food protein intake [25]. In addition, recent studies indicate that free EAA mixture supplementation promotes both more muscular protein synthesis and the expression of plasma anabolic and anti-inflammatory proteins than nutrition with whey proteins [26,27,28]. These observations could have several explanations.

Firstly, food proteins must be digested by pancreatic enzymes and the resulting AAs would then be absorbed by the intestine and introduced into the bloodstream to be transported to cells. As recently shown in aged and/or diseased patients’ pancreas, exocrine efficiency and intestinal metabolism are progressively reduced with consequently altered digestion and the absorption of food components. On the contrary, free AAs do not need to be digested, but are rapidly absorbed and immediately available in the blood for protein syntheses [20,26].

Secondly, from a nutritional point of view, AAs are classified as EAAs, which cannot be synthetized in the body and are therefore needed in the human diet, and non-essential (NEAAs) which can be produced in the body according to the metabolic need, so that their presence in the diet is not strictly necessary. Unfortunately, we should note that no dietary proteins have an EAA/NEAA ratio >0.9; whereas, conversely, most proteins have a <0.7 ratio at best.

Interestingly, experimental data show that special EAA mixtures with an EAA/NEAA ratio >1 increase the lifespan [29] and albumin [11], and also reduce inflammation in healthy mice [30,31]. This suggests that NEAAs are not indispensable for cell life and are synthesized according to metabolic needs if adequate amounts of EAAs are provided. Indeed, only EAAs have the metabolic characteristics illustrated below.

Only EAAs counteract IRS effects by activating glucose transport and protein synthesis [18]. These effects occur because EAA mixtures flowing through the portal vein are the signal for IGF-1 (insulin-like growth factor-1) secretion [18], which is the somatomedin responsible for activating anabolic growth hormones (GH) [18]. Interestingly, it has also been demonstrated that some specific AAs, such as leucine, directly stimulate proteins synthesis via sestrin2, which is a mTOR-regulating enzyme [32].

Additionally, data demonstrate that leucine’s ketoacid, referred to as beta-hydroxy-beta-methyl butyrate (HMB), as well as EAA mixtures, directly influences protein synthesis, stimulating the regulatory intracellular mTOR system [33,34,35]. However, it has been demonstrated that only EAA mixtures formulated according to human needs improve energy production and the synthesis of oxygen free radical scavengers through mitochondrial biogenesis [10]. In addition, EAA mixtures, but not ketoacids (i.e., HMB) and/or certain individual free AAs (i.e., leucine) can provide a sufficient concentration of AAs to support protein synthesis and provide an adequate amount of nitrogen essential for nitrogenous base production. These are an indispensable part of ATP and/or of NAD-NADH synthesis, which are crucial to maintain cellular redox homeostasis [10]. Finally, EAAs can also influence the insulin effects on adipocytes, enhancing glucose transport and modulating the use of FFAs [14].

Indeed, EAAs and their metabolites have recently been defined as “Metabokine” because they are able to influence local metabolism and systemic physiology [36], not only through direct metabolic influence, but also modifying the gene expression via epigenetic action [37]. Indeed, it has been shown that monocarboxylic acid, branched-chain essential amino acid (BCEAA) derivatives, 3-methyl-2-oxovaleric acid (MOVA), B-hydroxy-isobutyric acid (BHIBA) and amino acid 5-oxoproline (5OP) regulate adipocyte and myocyte metabolic gene expression of the enzymes involved in fatty acid oxidation. Moreover, they exert a transcriptional regulation of peroxisome proliferator-activated receptor-gamma coactivator-1alpha (PGC-1α), which is a transcriptional coactivator that regulates the genes involved in mitochondrial biogenesis and the adaptive metabolic response to exercise [38].

Because of all these points, we can conclude that oral supplementation with special mixtures of free EAAs, in a stoichiometric ratio and formulated according to human metabolic needs, should be used in CHF patients to contrast protein calorie malnutrition and sarcopenia, muscular wasting and cachexia. However, although the combination of exercise and nutritional support with AAs has solid rational foundations, the relationship itself is very complex. Experimental and clinical data available are quite contradictory and controversial. These mixed results can be partly interpreted by taking into account the experimental models used as the type of exercise (resistance or force), the different nutritional supplementations (food proteins, AA mixtures, ketoacids with or without micronutrients such as vitamins and/or ions), the age of the patients studied, as well as the presence of comorbidities [39].

It should be emphasized that nutritional therapies may substantially influence human metabolism. Human metabolism is a complex phenomenon characterized by chemical changes that take place in the cell following an intricate network of specific metabolic pathways, which govern physical processes that determine the cell physiology and biochemical properties.

EAA-related metabolism is the sum of all chemical reactions in which one specific chemical compound is transformed into other molecules through a series of steps. Each step is facilitated by a specific co-factor (i.e., vitamins, ions and others). The consequence of nutritional metabolic therapeutical approaches is that nutrition should provide not only one molecule, but all the most fundamental molecules involved in the anabolic EAA-mediated pathways [14,40].

This approach has been confirmed in our recent clinical study. This showed that a specific mixture of EAAs, efficient to match human metabolic needs, has the best effects in patients with CHF-induced hypercatabolic syndrome with proteins disarrangement (anaemia) when it is administered with co-factors (vitamin D, B6 and B9, iron) fundamental for the activation of the anabolic pathways of haemoglobin synthesis [4].

Moreover, recent data show that altered intestinal function, such as dysbiosis, and increased permeability are present in patients with CHF, and they negatively affect patient nutrition [16]. A recent exhaustive review paper supports our perspective. It analyses the effect of different micronutrients (i.e., Q10, L-arginine, antioxidants, vitamins A-C-D-E, ions and others), which have been proposed as influencing cardiovascular risk. Evaluation of the literature indicates that not all nutritional molecules are equal, given that the needs of patients may be different. As a result, we need to provide more personalized and specifically integrated dietary interventions involving combinations of beneficial supplements in adequate amounts, according to the specific metabolic needs of each patient [41]. Last but not least, it is necessary to distinguish between being fed with or without nutritional supplementations, and nutrition itself.

Being fed means furnishing food and oral supplementation for the maintenance/improvement of the body’s metabolism. On the contrary, nutrition is the sum of biochemical and physiological complex processes by which a human being digests, absorbs and metabolizes the macro-micronutrients introduced via ingestion. These data and observations suggest that personalised, functional and integrated nutritional therapies, which consider all the salient aspects of nutrition and exercise, should be used to perform the best and avoid contrasting data.

## 4. Molecular Hypothesis of Exercise Training and Nutritional Supplementation Alliance: The Roles of AMPK and mTOR

During exercise, cells consume energy-splicing adenosine tri-phosphate (ATP), which is split off from one of its three phosphates, becoming ADP (adenosine di-phosphate) and phosphates. Likewise, energy is also released when a phosphate is removed from ADP to form adenosine monophosphate (AMP). Interestingly, increasing AMP concentrations stimulates AMP-activated protein kinase (AMPK), an enzyme which acts as an energy sensor and activates a process called autophagy (A). A is characterized by the breakdown and recycling of the components of aged macromolecules, including proteins and lipids, promoting substrate availability for energy production [42].

It is well known that physical exercise induces A. However, exercise-induced A is not totally negative because it allows for the energy production fundamental for the preservation of energy production and mitochondrial functions during muscular contraction. Therefore, it has been postulated that A induced by exercise is an adaptive response that avoids mitochondrial damage in turn, induced by physical activity due to energy deficiency caused by the reduced availability of energetic substrates during exercise [43]. To modulate energy expenditure/production, AMPK also spares energy by blunting the multi-enzymatic system, mammalian or mechanistic target of rapamycin (mTOR) [42].

mTOR is a multi-enzymatic complex which regulates many fundamental processes of cell life (i.e., protein synthesis, autophagy and others), integrating external (i.e., nutrients such as EAAs, growth factors and others) and internal stimuli (energy reduction). It is intuitive that the mTOR system is a central regulator of mammalian metabolism and physiology, with important roles in the regulation of cell functions such as energy production and protein synthesis, cell survival and duplication, integrating stimuli through complex molecular signaling, which is not yet fully understand.

mTOR is formed mainly of two distinct complexes, mTORC1 and TORC2. These two sub-units contain other regulatory proteins (Raptor, Protor PRAs40, Rictor), most of which act as the active repressors of mTORC activity. Both complexes may be inactivated by a subunit called DEP domain-containing mTOR-interacting protein, named Deptor [44]. Indeed, an elevated cell concentration of AMPK inhibits mTORC1-dependent protein synthesis and activates A to make energetic molecules available. On the contrary, A is blocked when AMPK decreases and cellular ATP increases.

Interestingly, high levels of ATP stimulate protein synthesis via mTORC1 activation. It is important to understand that EAAs (particularly leucine and the metabolically derived glutamine) stimulate TORC1-mediated protein synthesis (i.e., sestrin), mitochondrial biogenesis and increment energy production by providing both carbon skeletons and Krebs Cycle intermediates. As described before, the anabolic process is a complex phenomenon with many processes, such as autophagy, energy and substrate (especially EAA) availability, mitochondrial function and AMPK concentration. These processes interact with each other and should be perfectly orchestrated.

Although these mechanisms are not yet fully understood, it is becoming increasingly obvious that the anabolic process is particularly activated when there is: (1) a high EAA concentrations (especially leucine), able to stimulate proteins synthesis via mTORC1, (2) an increased function of sestrin, which is able to activate mTORC1, and (3) a decreased function of mTORC1 inhibitor, Deptor [45].

These observations are demonstrated in a very elegant experimental animal research paper, which shows that only an immobilised limb, with probable conserved energy and therefore reduced AMPK, has mTOR resistance to nutrient stimulation when animals are fed. Indeed, the enhanced function of Deptor and decreased synthesis of sestrin due to immobilization, block the nutrient (EAA)-dependent anabolic pathway via mTORC1 [46].

**Figure 1:**
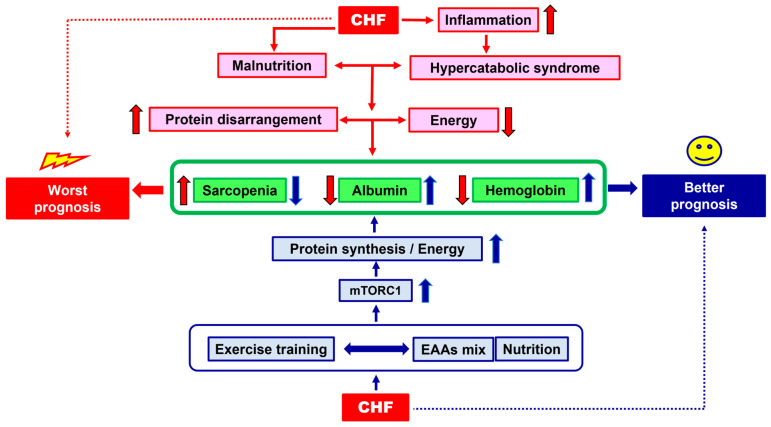
Schematic representation of the effects of the alliance between exercise training and nutritional EAA supplementation on CHF prognosis. Red arrows = the way to a worst prognosis; blue arrows = the way to a better prognosis. Arrow up = increase; arrow down: decrease.

The effects of the alliance between exercise training and nutritional EAA supplementation on CHF prognosis is schematized in Figure 1.

## 5. Conclusions and Clinical Suggestions

Nutritional supplementation with a mix of all free EAAs and co-factors of anabolic pathways (if found reduced and/or altered), concomitantly with physical exercise, could be allies in the treatment of CHF and reinforce the effects of standard medical therapy. According to the molecular and pathophysiological evidence, we propose a road map to turn nutritional supplementation and physical activity into an effective alliance.

Firstly, the presence of protein energy malnutrition with signs of sarcopenia and globular proteins disarrangement (i.e., through anthropometric measurements and blood albumin quantification, etc.) in CHF patients should be looked for and evaluated.

**Figure 2:**
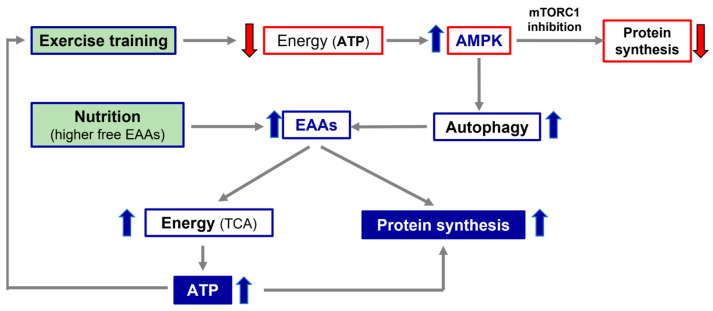
Simplistic representation of the importance of exercise and nutrition in promoting protein synthesis and energy production. TCA, tricarbossilic acid cycle; ATP, adenosine tri-phosphate; AMPK, AMP-activated protein kinase; EAAs, essential amino acids.

Secondly, the alteration of the processes interfering with a patient’s optimal nutrition, such as intestinal dysbiosis or leaky gut syndrome, as well as possible deficiencies of the molecules involved in the conservation of anabolic pathways (i.e., vitamins, ions), should all be evaluated. If nutrition and/or metabolic molecules are altered and/or reduced, specific therapies should be initiated and continued until their functions and/or concentrations reach normal values. The pivotal role of the alliance of exercise and nutrition with higher free EAA availability, in promoting protein synthesis and energy production, is illustrated in Figure 2.

Simultaneously, both nutritional supplementation with specific mixtures of all free EAAs tailored to human needs, and personalized physical exercise based on existing protocols, should be initiated. Further clinical studies are needed to confirm and ameliorate our suggestions.

## Data Availability

Not applicable.
